# Design and Construct an Optical Device to Determine Relative Blood Volume in Patients Undergoing Hemodialysis

**DOI:** 10.5812/ircmj.15603

**Published:** 2014-04-05

**Authors:** Banafshe Dormanesh, Shahnaz Tofangchiha, Vahid Abouei, Hani Sharifian

**Affiliations:** 1 Department of Pediatric Nephrology, AJA University of Medical Sciences, Tehran, IR Iran; 2 Department of Internal Medicine, AJA University of Medical Sciences, Tehran, IR Iran

**Keywords:** Blood Volume, Hypotension, Renal Dialysis, Optical Devices, Blood

## Abstract

**Background:**

Occurrence of hypotension during hemodialysis in nearly 20-30% of patients, shows is the necessity of continuous monitoring the patients' blood pressure during hemodialysis. Since directly and non-invasively continuous blood pressure monitoring, is not easy, finding a parameter related to blood pressure, for indirect monitoring is of great value. Related blood volume (RBV) is one of the parameters, related to blood pressure and have a good potential to reflect the patient’s hemodynamic condition.

**Objectives:**

The main objective of this study was to design and construct an optical device to determine the RBV in patients undergoing hemodialysis, during the process.

**Materials and Methods:**

After initial studies in order to select a proper sensor, using the ORCAD software, an analog circuit was designed. The implementation and modification of the circuit was done by the clinical tests, using expired blood. Afterwards, for calculation the RBV, controlling the display, data storage and sending it to the computer, an ATmega16 microcontroller was used. For programing the microcontroller, CodeVision software and then Altium Designer software were used for the circuit compression, in order to design the printed circuit board. Finally, all parts of the analog and digital circuit, AC to DC converter and the LCD were embedded in a box.

**Results:**

After finalization of the device and before testing it in a real situation, expired blood was used for final evaluation. The evaluation was done by changing the blood concentration, at the start point by adding water to it. In fact, the device can track the changes in blood concentration and display the RBV. After this evaluation, the device was tested in a clinical situation. The results showed there are no interactions between this device and the other devices used in the dialysis section and it can work properly in order to measure the RBV.

**Conclusions:**

Considering the hypotension and its consequences in a patient on hemodialysis, solving this problem seems necessary. One method for preventing this, is to use the blood pressure related parameters and one of these parameters is the RBV. In this study, in order to measure the RBV, a device was designed and evaluated by expired blood and also tested in a clinical situation. Results showed that the device could work properly in order to measure the RBV.

## 1. Background

Ultrafiltration rate of hemodialysis machines should be adjusted in a way that the fluid taken from the blood be compensated by the interstitial liquid, otherwise the blood volume may be decreased and the patient may experience hypotension and its complications. This problem occurs in about 20-30% of the patients on hemodialysis. To prevent this incident, it is necessary to continuously monitor the patients' blood pressure during the hemodialysis session (1).

Continuous, non-invasive and direct monitoring of blood pressure is not easy (1, 2). Direct observance of blood pressure by a nurse is not possible and the highest frequency with which a nurse can measure each patient's blood pressure, is nearly every 15 minutes. Therefore, in order to solve this problem, it is necessary to use the parameters associated with blood pressure or blood volume in patients on dialysis. Until recently, different methods has been suggested to determine parameters related to patients' blood pressure, some synchronously measured and used in the control process for the optimal performance of hemodialysis and some calculated asynchronously and used for the future treatments. One of the parameters related to blood pressure is the related blood volume (RBV) and there have been devices invented to measure this parameter in order to prevent hypotension in patients on hemodialysis, during the dialysis (1, 3).

The attractiveness of the non-invasive RBV tracking devices is based on the synchronous, continuous and repeatable nature of their work in measuring the RBV, during the dialysis sessions. This non-invasive technique is based on the concentration of the blood components. The blood concentration changes by the change of plasma volume (1, 4).Changes in the RBV are calculated by Equation 1:

Equation 1. $$\mathrm{\Delta RBV}(\%)=((\frac{C_{0}}{C_{t}})-1)\times 100$$

C_0_ and C_t_ are blood component concentrations at the start point and at a particular point of time, during the dialysis.

So far, different methods of blood volume monitoring including optical, electrical and mechanical methods are invented and developed (4). RBV measurement devices use hemoglobin (Hb), hematocrit (Ht) (1) or the total protein of plasma concentration in order to measure the RBV (1, 4).

Some studies (with a maximum number of 11 patients) have also shown that setting UFR according to the RBV changes have a positive effect on prevention of hypotension (1, 5). Surprisingly, in one study, RBV was shown to be the most powerful predictive parameter to prevent hypotension during dialysis (1).

In another study, as a new method for monitoring blood volume changes (hypertension), blood viscosity was used. In theory, the viscosity is related to A-V pressure gradient during dialysis. Blood viscosity (calculated by Hugen-Poiseuille formula), is related to blood flow and arterial pressure (A) before pumps and venous pressure (V) changes. The A-V pressure gradient fluctuates with pump rotary vibrations, therefore to minimize the noise, the A-V pressure gradient is measured in certain points of pump rotary. This simple and inexpensive method makes it possible to monitor blood volume changes, and therefore provides data for controlling fluids in patients on dialysis and prevent occurrence of hypotension (6). Based on the tests, it was found that the A-V pressure gradient (ΔP) is independent from flow rates, up to 300 mL/min, therefore the pressure gradient can be corrected through the Equation 2 for different flow rates.

Equation 2. $$\Delta P_{c}=\Delta P_{\alpha }\times (\frac{Q_{b}}{Q_{a}})$$

Where corrected A-V pressure gradient (ΔP), is A-V pressure gradient after blood flow changes, is blood flow rate before change, and is blood flow rate after change. In another method, a system is designed to monitor the oxygen saturation in venous blood line of hemodialysis machine, as a marker of hypotension. Oxygen saturation monitoring, can show the decrease in cardiac output. The reduction in cardiac output happens prior to the occurrence of hypotension, which makes it possible to predict the hypotension through this method (7). Heart rate can be estimated from the arterial pressure signal sent by the dialysis machine, without using ECG. One indicator of hypotension is the changes of heart rate and to avoid hypotension, getting information about heart rate and its changes is helpful. For this purpose, the arterial or venous pressure signals within the dialysis machine are used. Afterwards the main issue is separating the very weak heart rate signal, induced by the strong blood pump signal. To extract the heart rate signal, the measured signal should be changed to the frequency domain, with the use of FFT and Blackman-Harris window. The heart rate signal amplitude is very small, in the range of 1 Hz, but if the frequency difference between the first peak of heart rate and blood pumping be more than 0.15 Hz, it is possible to separate two signals through the signal separation technique (8).

Among all the methods developed to determine these parameters, some are more practical, having different uses and procedures to control the dialysis process.. Some methods, use the parameters to automatically control the process but, in some other, parameters are measured only to warn nurses to control the dialysis machine manually. Crit-Line system is made by Hema Metrics as an independent machine. This system uses a non-invasive optical method and measures the absolute value of Ht and oxygen saturation and through this, calculates the RBV changes, during the hemodialysis process (2). Hemoscan developed by Gambro-Hospal, uses another method and is installed on the dialysis machine, to function as a single device. This system measures the amount of Hb and calculates the changes of theRBV, according to the blood optical absorption (2, 9, 10).

Blood volume monitor (BVM), is developed by Fresenius and is installed on Fresenius 5008 dialysis machine. This system works by measuring the total protein concentration (TPC), with ultrasonic method (2, 11). The BVT system calculates the actual amount of the RBV and adjusts UFR and dialysate conductivity, during hemodialysis. Many studies have shown that hemodialysis by BVT has more dynamic blood stability, compared to the standard hemodialysis (1, 12).

## 2. Objectives

The main objective of this study was to design and construct an optical device to determine the RBV in patients undergoing hemodialysis, during the process.

## 3. Materials and Methods

**Figure 1.**
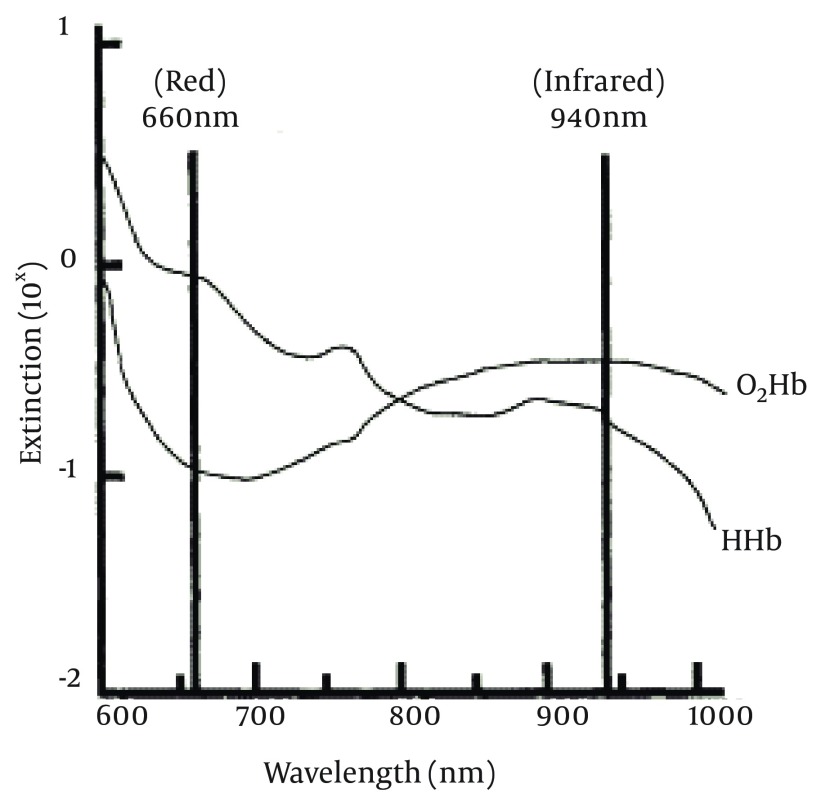
The Absorption Coefficient of Two Different Types of Hemoglobins; Oxyhemoglobin (HbO_2_) and Reduced Hemoglobin (HHb) (13)

Regarding the relationship between the RBV and hypotension in patients undergoing hemodialysis, the authors decided to design and construct a device to measure the RBV. After initial studies, in order to select a proper sensor, using ORCAD software an analog circuit was designed. The implementation and modification of the circuit was done by the clinical tests, using expired blood. Afterwards, for calculation of the RBV, display controlling and data storage and sending it to the computer, an ATmega16 microcontroller was used. For programing the microcontroller CodeVision and then Altium Designer softwares were used for the circuit compression, in order to design the printed circuit board. Finally, all parts of the analog and digital circuit, AC to DC converter and the LCD were embedded in a box. Real condition evaluations of this device show its ability to measure the RBV. Considering the optical sensors are inexpensive and more available, the optical method was used in order to measure the blood concentration. In hemodialysis process, blood plasma and tiny components in blood are exchanged by the dialysate and reduce by ultrafiltration. Other large components like Hb and other blood proteins do not get filtered during the hemodialysis process. By reducing the blood plasma, the protein concentration in the blood increases and consequently the optical absorption of blood increases. Due to the availability of 920 nm optical waves which are also used in pulse oximetry sensors and have an equal absorption coefficient for both oxyhemoglobin (HbO_2_) and reduced Hb (HHb) (the main Hb forms in the blood), this type of optical sensor was used in the present study (Figure 1).

**Figure 2.**
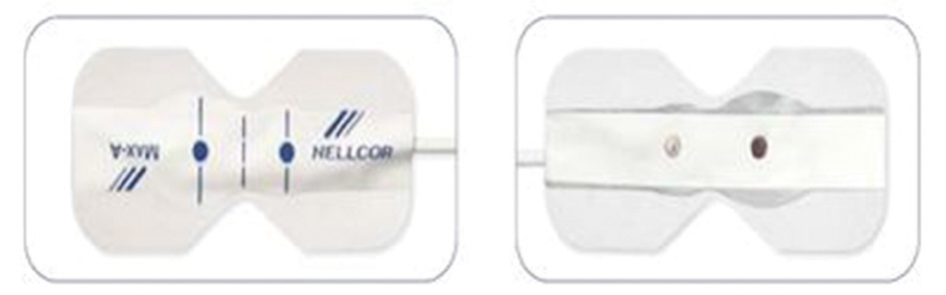
Nellcor Sensor D-25 (14, 15)

**Figure 3.**
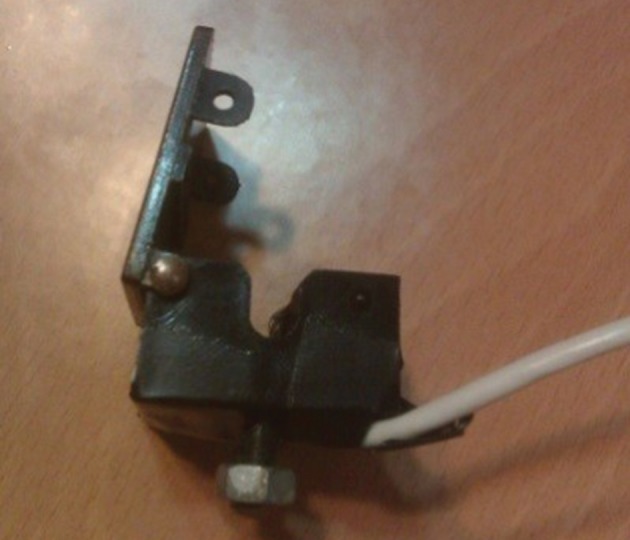
Prepared Sensor for Measuring the Blood Concentration

For this purpose, the model D-25 sensor, manufactured by Nellcor Company (Figure 2) was used. This sensor should be ready to be installed on dialysis tube set. To do this, infrared transmitter and receiver were placed inside the frame of ultrasonic sensors. As shown in Figure 2, the frame has a particular space for the dialysis tube set. It should be noted that for the test, the sensor is placed before the arterial route on the dialysis set (Figure 3). For measuring the blood concentration changes, a sensor should be placed on the dialysis set. Depending on the dialysis set, the pipe material and its thickness, the received signal may have a constant DC level with different values. Due to the dependence of the absolute value of the DC level, on the physical characteristics and environmental conditions, it is not of importance in the present study's calculations.

**Figure 4.**
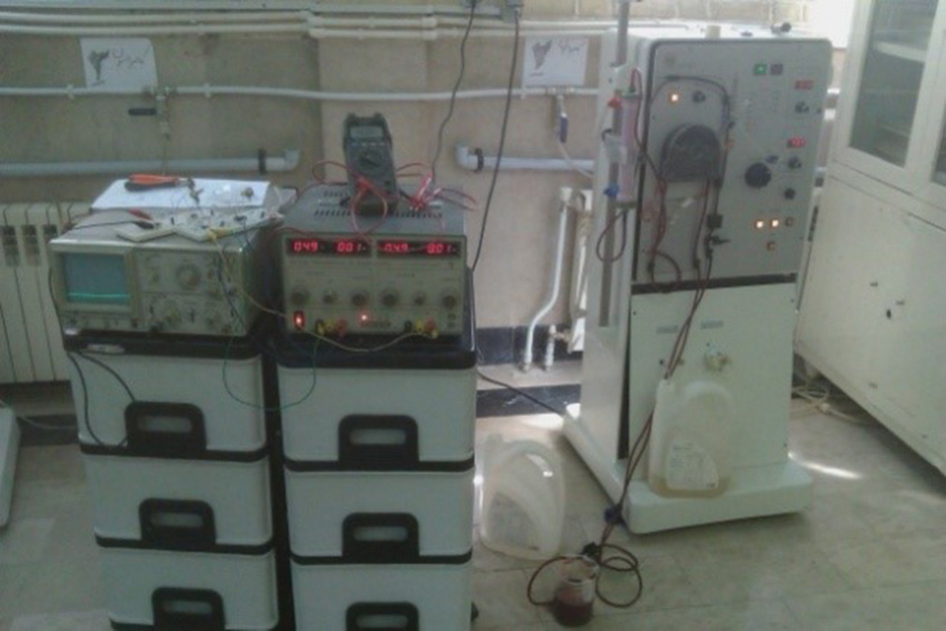
Modification of the First Circuit Using Expired Blood

**Figure 5.**
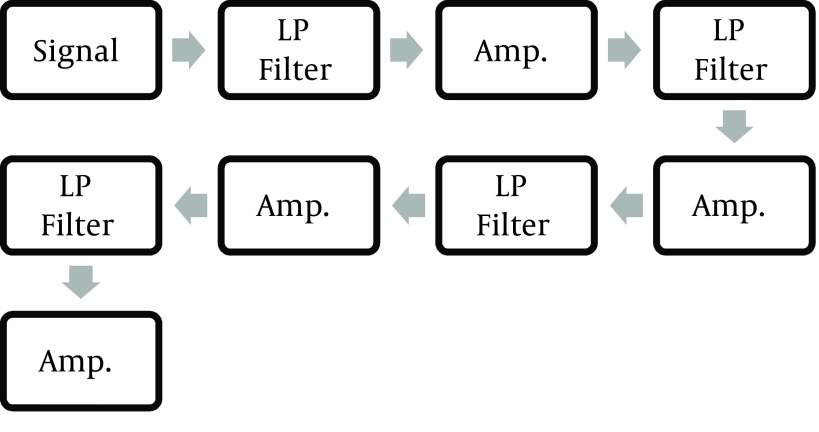
Block Diagram of the Final Analog Circuit

To track the changes in blood concentration, it is important to notice the changes in the DC signal. Using the ORCAD software, the first analog circuit was designed to measure the RBV. After first implementation, the circuit was modified through trial and error. After using the expired blood and changing its concentration, the final modification was done. The block diagram of the final analog circuit is demonstrated in Figures 4 and 5. Due to the weakness of the received signal, a high gain amplifier was essential to proceed the studies. According to the DC value of the original signal and after its filtering, the signal was amplified to an extent that the op-amps were not saturated. Simultaneously with amplifying the signal by a potentiometer, the DC amount of amplified signal was reduced, until fluctuation of the signal reached 0 V. In the final circuit, four layers of these filters and amplifiers with different gains and outputs were used. Output of each amplifiers is available to calibrate the device, depending on the needs. In the designed filters, due to the non-periodic nature of the main signal, the filter cutoff frequency of 10 Hz was considered, in order to eliminate the noises on the AC current (220 Hz). Considering these, the calculation of required resistance and capacity for each filter was calculated through the Equation 3.

Equation 3. 10=12πRC→RC≅16×10-3{R=150 kΩC=10μF

**Figure 6.**
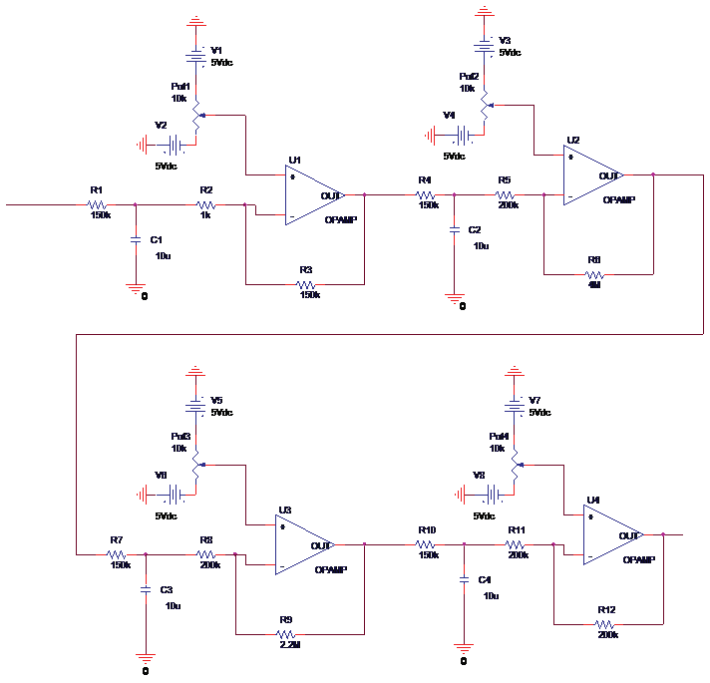
Final Analog Circuit for RBV Measurement; Input Comes From the Optical Sensor and Output goes to the Digital Circuit

**Figure 7.**
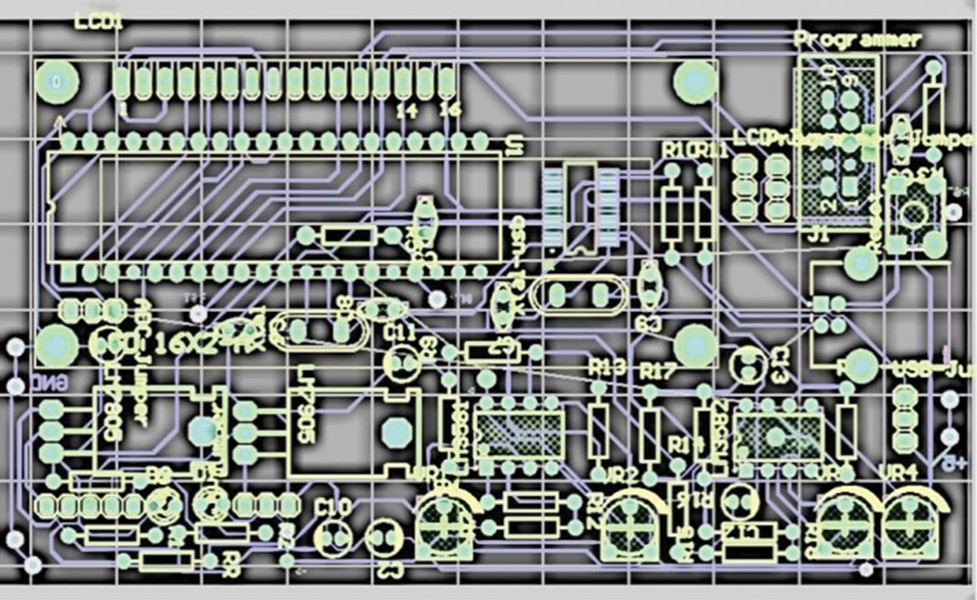
Final Pcb

**Figure 8.**
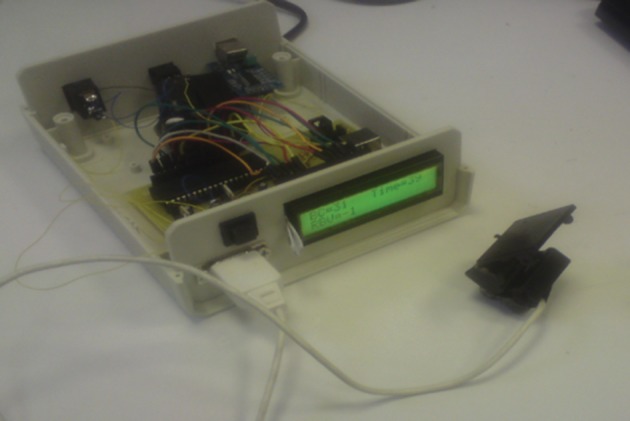
Embedment of All Parts in a Box

**Figure 9.**
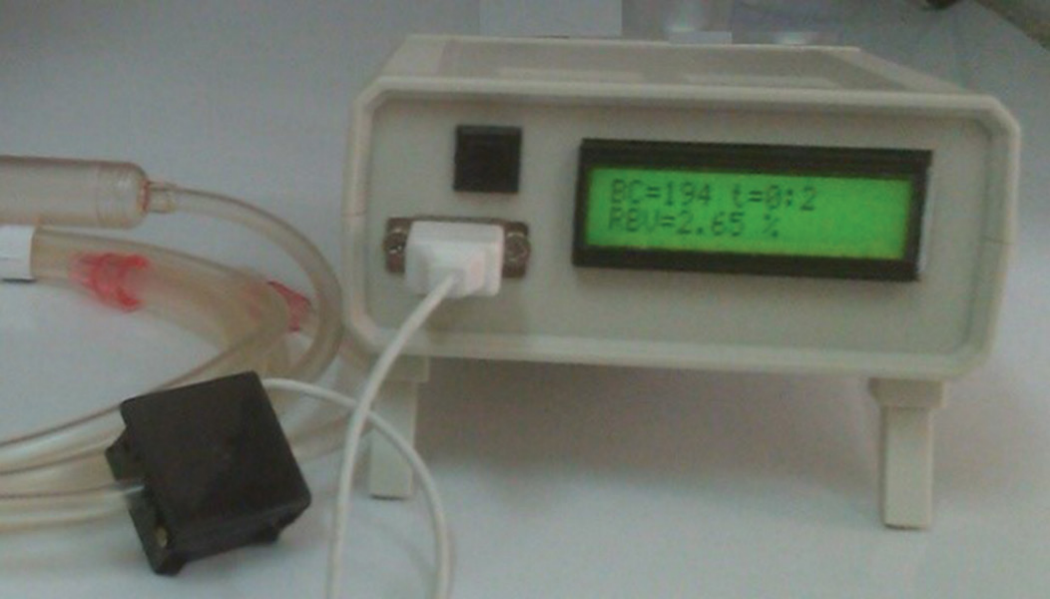
An Optical Device for RBV Measurement

The final analog circuit is demonstrated in Figure 6. As it is shown, the used op-amp was LM358. For the RBV calculation, control of LCD, data storage and sending the data to the computer, digital circuit was designed by ATmega16 microcontroller. The program for this microcontroller was written by CodeVision software. After finalization of the two circuits (analog and digital) for all their different parts to integrate, Pcb was designed, using the Altium Designer software as demonstrated in. After designing the Pcb board and embedment of all components of the two circuits on it, a box was used for embedding all parts, like the Pcb board, LCD, serial port, On-Off switch, sending data switch and AC to DC convertor. Finally, the device was prepared to work in real situation, for clinical studies. As demonstrated in Figure 8, the device can measure and display the RBV, in addition to displaying the duration of the dialysis (Figures 7-9).

## 4. Results

**Figure 10.**
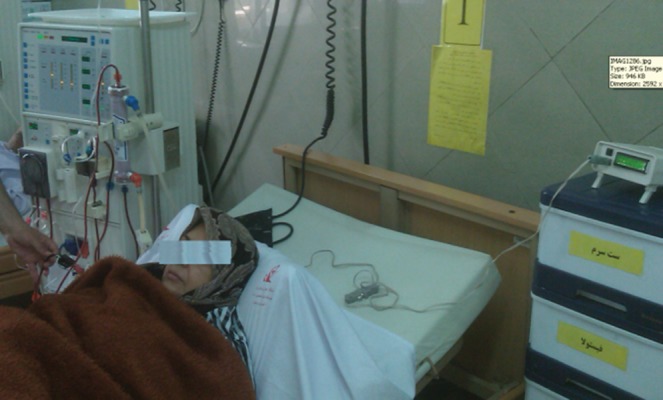
Testing the Device in a Clinical Situation

After finalization of the device and before testing it in a real situation, expired blood was used for the final evaluation. This evaluation is done by changing the blood concentration from the beginning, by adding water to it. In fact, the device can track the changes in blood concentration and display the RBV. After this evaluation, the device was tested in a clinical situation as shown in Figure 9. The results showed that there are not any interactions between this device and the other devices functioning in the dialysis section and it can work properly in order to measure the RBV. To produce this device, one of our purposes was to evaluate the relationship between RBV and blood pressure patients undergoing dialysis, a purpose which is being studied in the same authors' new survey (Figure 10).

## 5. Discussion

Continuous, non-invasive and direct monitoring of blood pressure is a big problem, and to do so it is necessary to find some parameters associated with blood pressure or blood volume. RBV is a good parameter, associated with the patient's blood pressure during the dialysis. In this regard, a device was designed with the ability to measure the RBV. To do this, as the first step, an analog circuit was designed, using the ORCAD software. For its evaluation, after first implementations, modifications were done with expired blood through trial and error. After finalization of the analog circuit, ATmega16 microcontroller was selected and used to design the digital circuit using the CodeVsion software. In order to integrate the whole parts of two circuits, Pcb was designed by the use of Altium Designer software and embedment of all components was done on it. Afterwards, a box was used, in order to embed all parts. The final device was tested in a clinical situation and the results showed that it can work properly for measuring the RBV. The authors are trying to find the quantitative correlations between blood pressure and the RBV, using the same device, in a new clinical study.
